# Aberrant SWI/SNF Complex Members Are Predominant in Rare Ovarian Malignancies—Therapeutic Vulnerabilities in Treatment-Resistant Subtypes

**DOI:** 10.3390/cancers16173068

**Published:** 2024-09-03

**Authors:** Yue Ma, Natisha R. Field, Tao Xie, Sarina Briscas, Emily G. Kokinogoulis, Tali S. Skipper, Amani Alghalayini, Farhana A. Sarker, Nham Tran, Nikola A. Bowden, Kristie-Ann Dickson, Deborah J. Marsh

**Affiliations:** 1 Translational Oncology Group, School of Life Sciences, Faculty of Science, University of Technology Sydney, Ultimo, NSW 2007, Australia; yue.ma-7@student.uts.edu.au (Y.M.); natisha.field@student.uts.edu.au (N.R.F.); tao.xie-1@student.uts.edu.au (T.X.); sarina.briscas@alumni.uts.edu.au (S.B.); emily.g.kokinogoulis@alumni.uts.edu.au (E.G.K.); tali.s.skipper@student.uts.edu.au (T.S.S.); amani.alghalayini@uts.edu.au (A.A.); amy.sarker@uts.edu.au (F.A.S.); kristie-ann.dickson@uts.edu.au (K.-A.D.); 2 School of Biomedical Engineering, Faculty of Engineering and IT, University of Technology Sydney, Ultimo, NSW 2007, Australia; nham.tran@uts.edu.au; 3 Drug Repurposing and Medicines Research Program, Hunter Medical Research Institute, Newcastle, NSW 2289, Australia; nikola.bowden@newcastle.edu.au; 4 School of Medicine and Public Health, University of Newcastle, Newcastle, NSW 2289, Australia

**Keywords:** SWI/SNF chromatin-remodelling complex, BAF chromatin-remodelling complex, ARID1A, ARID1B, SMARCA4, SMARCA2, ovarian clear cell carcinoma (OCCC), endometrioid ovarian cancer (EnOC), Small cell carcinoma of the ovary, hypercalcaemic type (SCCOHT), endometriosis

## Abstract

**Simple Summary:**

The rare ovarian cancer subtypes ovarian clear cell carcinoma (OCCC) and Small cell carcinoma of the ovary, hypercalcaemic type (SCCOHT), can have mutations in members of a complex known as SWI/SNF that regulates the accessibility of chromatin to factors involved in DNA repair and gene expression. Some mutations in a particular complex member also occur in endometrioid ovarian cancer (EnOC) and endometriosis. Patients with endometriosis have a greater risk of developing OCCC and EnOC, with endometriosis frequently present at the time of diagnosis of these malignancies. The OCCC and SCCOHT ovarian cancer subtypes are notoriously difficult to treat with chemotherapies based on platinum-drugs that are standard-of-care for most cases of ovarian cancer. Mutations in members of this chromatin-remodelling complex offer new opportunities for molecular therapeutics using drugs that inhibit different aspects of cellular processes, including DNA repair, epigenetic regulation, kinase activity, and immune checkpoints.

**Abstract:**

SWI/SNF (SWItch/Sucrose Non-Fermentable) is the most frequently mutated chromatin-remodelling complex in human malignancy, with over 20% of tumours having a mutation in a SWI/SNF complex member. Mutations in specific SWI/SNF complex members are characteristic of rare chemoresistant ovarian cancer histopathological subtypes. Somatic mutations in *ARID1A*, encoding one of the mutually exclusive DNA-binding subunits of SWI/SNF, occur in 42–67% of ovarian clear cell carcinomas (OCCC). The concomitant somatic or germline mutation and epigenetic silencing of the mutually exclusive ATPase subunits SMARCA4 and SMARCA2, respectively, occurs in Small cell carcinoma of the ovary, hypercalcaemic type (SCCOHT), with *SMARCA4* mutation reported in 69–100% of SCCOHT cases and SMARCA2 silencing seen 86–100% of the time. Somatic *ARID1A* mutations also occur in endometrioid ovarian cancer (EnOC), as well as in the chronic benign condition endometriosis, possibly as precursors to the development of the endometriosis-associated cancers OCCC and EnOC. Mutation of the *ARID1A* paralogue *ARID1B* can also occur in both OCCC and SCCOHT. Mutations in other SWI/SNF complex members, including *SMARCA2*, *SMARCB1* and *SMARCC1*, occur rarely in either OCCC or SCCOHT. Abrogated SWI/SNF raises opportunities for pharmacological inhibition, including the use of DNA damage repair inhibitors, kinase and epigenetic inhibitors, as well as immune checkpoint blockade.

## 1. Introduction

Ovarian cancer spans a number of different histopathological subtypes, most of which have limited treatment options beyond surgical debulking and combination chemotherapy consisting of systemic platinum-based drugs including carboplatin and the taxane paclitaxel [1]. In the most common subtype, high-grade serous ovarian cancer (HGSOC), Poly (ADP-ribose) polymerase (PARP) inhibitors have been shown to increase both progression-free survival (PFS) and overall survival (OS) in the presence of molecular aberrations in genes such as *BRCA1* and *BRCA2* which encode proteins functioning in the homologous recombination repair (HRR) pathway [2,3,4,5,6]. Anti-angiogenics targeting vascular endothelial growth factor (VEGF), such as bevacizumab, are also in use, with clinical trials suggesting the benefit of combining bevacizumab with the PARP inhibitor olaparib [5,6]. Therapeutic targeting of mutations beyond those directly involved in the HRR pathway in ovarian cancer is yet to emerge into the clinic. This is an area attracting extensive interest, especially for treatment-resistant subtypes such as ovarian clear cell carcinoma (OCCC) and the rare Small cell carcinoma of the ovary, hypercalcaemic type (SCCOHT), both with a poorer prognosis compared to HGSOC [7,8].

The intersection of genetics and epigenomics to drive chromatin remodelling in malignancy provides further opportunities for both uncovering the fundamental mechanisms of gene regulation and identifying new therapeutic targets. The ATP-dependent chromatin-remodelling complex SWI/SNF (SWItch/Sucrose Non-Fermentable; also known as the BAF complex) is an important junction for these intersections, with aberrations in SWI/SNF complex members reported in around 20% of all human malignancies [9,10]. Mutations in *SMARCA4*, that encodes one of catalytic subunits of SWI/SNF, occur in around 5–7% of all human malignancies (reviewed in [11]). Similarly, mutations in *ARID1A* (AT-rich interactive domain-containing protein 1A) that encodes a DNA-binding subunit of SWI/SNF have been reported in ~6% of human malignancies [12].

**Figure 1:**
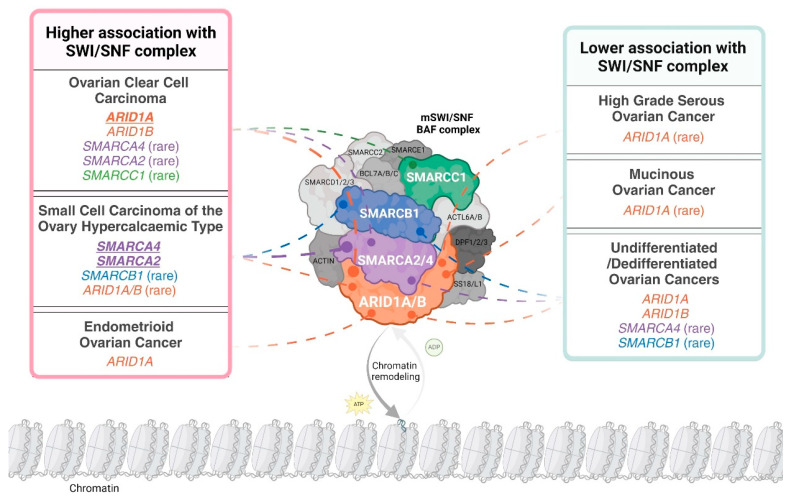
The distribution of loss-of-function alterations in mammalian SWI/SNF (mSWI/SNF) chromatin-remodelling complex members across ovarian cancer histopathological subtypes. The schematic uses the canonical BAF (cBAF) formation of the SWI/SNF complex to depict subunit involvement. A loss-of-function alteration is defined as the presence of a pathogenic mutation in the encoding gene and/or the loss of corresponding protein expression. “Higher association with the SWI/SNF complex” is defined as subtypes where over 20% of cases have alterations in at least one complex member. An exception was made for undifferentiated/dedifferentiated ovarian cancers due to limited incidence reporting. Complex members identified as altered in over 40% of a specific subtype are indicated in bold and underline. An alteration is presented as ‘rare’ if less than 10 cases were reported in the published literature or large cohort analyses (N ≥ 100) report an incidence less than 10%. Distribution is based on data in Tables 2 and 3 and Tessier-Cloutier and colleagues [30]. Created with www.BioRender.com, Access Date: 9 August 2024.

Subunits of the SWI/SNF complex are differentially mutated in distinct ovarian cancer subtypes. For example, *ARID1A* is frequently mutated in OCCC and endometrioid ovarian cancer (EnOC) but rarely mutated in HGSOC and mucinous ovarian cancer (MOC) [13,14,15,16]. *ARID1B*, the mutually exclusive paralogue of *ARID1A*, is mutated in OCCC [17]. Both *ARID1A* and *ARID1B* are rarely mutated in SCCOHT [18]. Interestingly, *ARID1A* is mutated in the benign condition endometriosis, with OCCC and EnOC described as endometriosis-associated ovarian cancers (EAOC) [14,19,20,21]. *SMARCA4* is the predominant SWI/SNF complex member mutated in SCCOHT, with mutations reported infrequently in OCCC and HGSOC [17,22,23]. Other subunits of SWI/SNF, specifically *SMARCA2*, *SMARCB1* and *SMARCC1*, are reported to be mutated at very low frequency in OCCC or SCCOHT [17,24,25]. The abrogation of SWI/SNF complex members is not only governed by genetic mutation, as SMARCA2 can be post-translationally silenced in SCCOHT [26] and more rarely in OCCC [26,27]. Mutations and epigenetic silencing of SWI/SNF complex members present therapeutic vulnerabilities, facilitating synthetic lethal approaches for the treatment of ovarian cancer [28,29]. Mutated or epigenetically silenced SWI/SNF subunits in ovarian cancer are depicted in Figure 1.

In this review, we outline the extent of SWI/SNF complex member mutations and epigenetic regulation in a range of histopathological subtypes of ovarian cancer. We show that mutations of SWI/SNF complex members predominantly occur in the less frequent and very rare subtypes of ovarian cancer that generally respond poorly to current standard-of-care therapies. Lastly, we discuss the therapeutic opportunities that mutations in SWI/SNF complex members may provide for the treatment of patients with these rarer subtypes of ovarian cancer.

## 2. SWI/SNF Chromatin-Remodelling Complex

SWI/SNF is critical to maintaining healthy cellular functions, including during embryonic development and for stem cell pluripotency [31,32,33], in the development of male and female gametes [34,35], in cell cycle control [36], and in the DNA damage response where this complex is rapidly recruited to double-strand breaks (DSBs) [37,38]. There are three distinct forms of the mammalian SWI/SNF complex, specifically canonical BAF (cBAF), polybromo-associated BAF (PBAF) and non-canonical BAF (ncBAF), consisting of both common and distinct complex members encoded by 29 genes [39]. These distinct forms each have up to 15 members constituting mammalian chromatin-remodelling complexes [32].

The SWI/SNF complex has significant roles in transcription via its ability to modulate DNA accessibility. This complex is highly enriched at gene enhancers that bind transcription factors, where it is associated with the active chromatin mark histone H3 lysine 27 acetylation (H3K27ac) and has roles in directing lineage specificity [40,41]. SWI/SNF complexes are also located at promoters and transcription start sites (TSSs) where they function to regulate transcription [42]. Additionally, these complexes are known to preferentially associate with chromatin enriched with the active histone mark of monoubiquitylated histone H2B at lysine 120 (H2Bub1), which is reliant upon the histone writer Ring Finger Protein 20 (RNF20) [43,44]. Of note, SWI/SNF has antagonistic roles in transcription with proteins from the Polycomb group (PcG) such as EZH2 (Enhancer of Zeste Homolog 2) as part of the PRC2 (Polycomb repressor complex 2) that is associated with gene silencing via the catalysis of the repressive mark trimethylation of histone H3 lysine 127 (H3K127me3) [45,46,47].

Here, we focus on SWI/SNF complex member genes and their proteins, that are either mutated or themselves epigenetically regulated in rare subtypes of ovarian cancer. Specifically, we will review *SMARCA4*, *SMARCA2*, *SMARCB1*, *SMARCC1*, *ARID1A* and *ARID1B*. SMARCA4 (SWI/SNF-Related, Matrix-Associated, Actin-Dependent Regulator of Chromatin, Subfamily A, Member 4) and SMARCA2 (SWI/SNF-Related, Matrix-Associated, Actin-Dependent Regulator of Chromatin, Subfamily A, Member 2) are mutually exclusive ATP-dependent helicases that are present in all of cBAF, PBAF and ncBAF. These catalytic subunits are critical to chromatin remodelling, requiring ATP hydrolysis to enable nucleosome sliding and histone eviction, modulating access to DNA [39,48,49].

Non-catalytic SWI/SNF complex members also have roles to play in ovarian tumorigenesis. cBAF has 12 complex members and is the only BAF complex to include the mutually exclusive AT-rich interaction domain (ARID)-domain containing proteins ARID1A and ARID1B that bind to DNA (also known as BAF250A and BAF250B (BRG/BRM-associated factors A and B) [39,50]. SMARCB1 (SWI/SNF-Related, Matrix-Associated, Actin-Dependent Regulator of Chromatin, Subfamily B, Member 1) is an evolutionarily conserved subunit of SWI/SNF. The coiled-coil structural motif of the C-terminal domain of SMARCB1 contains a basic alpha-helix that directly binds to the acidic patch region of nucleosomes and is critical to the facilitation of chromatin remodelling [51]. SMARCC1 (SWI/SNF-Related, Matrix-Associated, Actin-Dependent Regulator of Chromatin Subfamily C Member 1), is a chromodomain-containing protein and core member of SWI/SNF that works as a scaffold to promote the stability of the complex, likely by preventing the proteasomal degradation of key complex members [39,52]. For ease of navigating the literature, the alternative nomenclature that is used for the SWI/SNF complex members discussed in this review is summarised in Table 1.

When viewed as an entire complex, SWI/SNF is the most frequently mutated chromatin-remodelling complex in human malignancy, with around 20% of human cancers harbouring a mutation in one of the SWI/SNF complex members (reviewed in [39,53]). Of note, component genes of SWI/SNF can also carry heterozygous mutations in neurodevelopmental and autism spectrum-like disorders including Coffin-Siris Syndrome [OMIM: #135900], Nicolaides–Baraitser syndrome [OMIM: #601358], congenital hydrocephalus and Hirschsprung’s disease [39,54]. The clinical association of mutated SWI/SNF complex members with developmental disorders supports key roles for complex members in determining cell fate [55]. SWI/SNF mutations could present viable therapeutic targets, increasing the response to immune checkpoint blockade (ICB) therapy, likely due to the differential expression of genes that stimulate the immune response. This would appear to be applicable even in tumours with low mutational burden, traditionally thought to respond poorly to immunotherapies [53].

## 3. SWI/SNF Complex Members Are Primarily Mutated in Poorer Prognosis Ovarian Cancer Subtypes and Endometriosis

Distinct subtypes of the group of ovarian cancers that harbour mutations of SWI/SNF complex members in catalytic, DNA-binding and core-stabilising subunits have some commonalities. In comparison to HGSOC that occurs in around 70% of all ovarian cancers, SWI/SNF-mutated ovarian cancers occur more rarely, present at an earlier age, and are more chemoresistant. With the exception of SCCOHT where both germline and somatic mutations can occur, the mutations of genes encoding SWI/SNF complex members in ovarian cancer occur somatically (see Table 2). While highly aggressive dedifferentiated and undifferentiated ovarian carcinomas are not discussed in depth in this review, these rare malignancies are also reported to have lost the expression of key SWI/SNF complex members [30]. The mutation of *ARID1A* has also been observed in endometriosis, with evidence suggesting that endometriotic lesions are precursors to the development of OCCC and EnOC, with disease progression driven by loss of this tumour suppressor [56]. In the following, we focus on the clinical nature and presentation of SWI/SNF-mutated ovarian cancers and discuss the extent of SWI/SNF complex member mutations in these tumours.

### 3.1. Ovarian Clear Cell Carcinoma (OCCC)

#### 3.1.1. Clinical Presentation and Epidemiology of OCCC

The median age of diagnosis of patients with OCCC has been reported as 55 years, much lower than that for patients with the more frequently diagnosed serous ovarian cancers at 64 years [7]. OCCC is one of the rarer ovarian cancer histopathological subtypes, with reported differences in frequency based on race and geographical location. In North America and Europe, OCCC is reported to constitute between 1–12% of all ovarian cancers; however, in Asian countries, this frequency is higher [57]. In Korea, around 12.5% of patients with ovarian cancer have OCCC [58]. A study conducted at Qingdao University in China showed that 14.2% of women with ovarian cancer in a cohort of 697 patients from a single hospital had OCCC [59]. In Taiwan, around 20% of patients with ovarian cancer have been reported to have OCCC [60]. Japan demonstrates the highest frequency of OCCC patients, constituting almost 27% of all patients with ovarian cancer [61]. The Surveillance, Epidemiology, and End Results (SEER) study showed that 4.8%, 3.1%, and 11.1% of white, black, and Asian women, respectively, living with ovarian cancer in the USA, have the OCCC subtype [7]. The drivers of these global differences in frequency are currently unclear, although it is interesting to note that Asian women living in the USA have higher frequencies of OCCC compared to non-Asian women, suggesting that ethnicity does play a role in the development of this subtype.

OCCC exhibits distinctive histopathological characteristics and clinical behaviours compared to other ovarian cancer subtypes. Macroscopically, this tumour mostly presents as a unilateral mass that is either solid, a mix of solid and cystic, or a predominantly cystic mass. Microscopically, characteristic features often include a combination of solid, tubulo-cystic and papillary patterns, with cells having large nuclei and abundant cytoplasmic glycogen that give them a clear appearance [62,63,64,65,66]. Clear cell changes are also observed mixed with other ovarian cancer subtypes [66]. OCCC is frequently found in conjunction with endometriosis, with endometriotic lesions speculated to be the precursor lesion for this malignancy [56,67].

OCCC tends to be diagnosed at an earlier FIGO (International Federation of Gynaecology and Obstetrics) stage than HGSOC, with studies reporting between 48.5 and 56.3% of OCCC presenting at Stage I compared with 12–16.6% of serous ovarian cancers, and between 9.9–11% of OCCC at Stage II compared with 5.5–7.2% of serous tumours [7,68]. Conversely, when considering advanced stages, 20.9–30.7% of OCCC were reported as Stage III at diagnosis compared with 45.7–61.7% of serous tumours. Diagnosis with Stage IV disease occurred in only 10.9–11.8% of OCCC compared with 16.2–35.2% of serous tumours [7,68]. In a large meta-analysis, OCCC patients were shown to have a poorer prognosis relative to tumour stage than other ovarian cancer subtypes, especially when diagnosed with advanced disease [69]. Furthermore, it has been shown that when diagnosed at early stages, OCCC patients had better PFS times compared to patients with serous ovarian cancer; however, OS was significantly decreased for these patients compared to those with serous ovarian cancer [70]. Similar findings have been reported by others [59,71]. In the context of therapy, between 11–27% of women with OCCC respond to first-line platinum-based therapy, with only 1–2% of women responsive to treatment once relapse has occurred [68,72,73]. These studies clearly indicate that there would be benefits to diagnosing OCCC in patients at an earlier stage; however, as for all ovarian cancer, OCCC currently lacks early screening tests that would enable this goal.

#### 3.1.2. SWI/SNF Complex Member Mutations in OCCC

Of the SWI/SNF complex members, *ARID1A* is the most frequently mutated in OCCC, with studies reporting between 42–67% of these tumours with somatic *ARID1A* mutation (Table 2) [13,14,15,17,74,75,76,77,78,79]. The immunohistochemical loss of ARID1A is seen in between 15–76% of OCCC (Table 3) [15,27,80,81,82]. *ARID1A* behaves as a tumour suppressor gene in OCCC, with both alleles presumed to be affected by concomitant variants, specifically mutations (deletions and insertions as well as nonsense mutations, all leading to premature STOP codons that would truncate the normal protein) and loss of heterozygosity, or the presence of presumably biallelic mutations [14,76]. In a study of 55 OCCC, the *ARID1A* paralogue *ARID1B* was reported to be mutated in 18% of tumours (Table 2) [17]. Tumours in which both *ARID1A* and *ARID1B* are mutated are reported to retain a wild-type allele of *ARID1B*, providing evidence that a certain level of wild-type ARID1B is essential to avoid synthetic lethality in *ARID1A*-mutant tumours [83]. An absence of functional ARID1A would seem to result in dependency on its mutually exclusive paralogue ARID1B, the depletion of which has been shown to destabilise SWI/SNF and inhibit cellular proliferation [84,85]. While this has yet to result in a new therapy for *ARID1A*-mutated tumours, the identification of a drug(s) that targets and abolishes ARID1B function in *ARID1A*-mutated tumours could represent a new therapeutic strategy for OCCC.

**Table 2:** Mutations in genes encoding SWI/SNF complex members identified in primary ovarian cancers of different histotypes.

Gene	Histotype	Mutated	Reference
*ARID1A*	OCCC	66.7% (32 of 48)	Shibuya et al., 2017 [13]
	OCCC	62% (24 of 39)	Murakami et al. [75]
	OCCC	57% (24 of 42)	Jones et al. [76]
	OCCC	55% (17 of 31)	Wiegand et al. [79]
	OCCC	49% (27 of 55)	Schnack et al. [78]
	OCCC	46% (55 of 119)	Wiegand et al. [14]
	OCCC	42% (23 of 55)	Itamochi et al. [17]
	OCCC	41.5% (17 of 41) *^a^*	Kuroda et al. [15]
	OCCC	7 of 9	Su et al. [74]
	OCCC *^b^*	1 of 1	Kihara et al. [77]
			
	EnOC	45% (9 of 20) *^a^*	Kuroda et al. [15]
	EnOC	30% (10 of 33)	Wiegand et al. [14]
	EnOC	21% (5 of 24)	Wiegand et al. [79]
	EnOC	1 of 7	Su et al. [74]
			
	HGSOC	19% (6 of 32)	Vaicekauskaitė et al. [16]
	HGSOC	0% (0 of 76)	Wiegand et al. [14]
	HGSOC	0% (0 of 36) *^a^*	Kuroda et al. [15]
			
	MOC	2 of 6 *^a^*	Kuroda et al. [15]
			
	SCCOHT	1 of 6	Auguste et al. [18]
	SCCOHT	1 of 1	Genestie et al. [86]
	SCCOHT	1 of 1	Sanders et al. [87]
*ARID1B*	OCCC	18% (10 of 55)	Itamochi et al. [17]
			
	SCCOHT	1 of 6	Auguste et al. [18]
	SCCOHT	1 of 1	Genestie et al. [86]
*SMARCA4*	SCCOHT	100% (12 of 12) *^c^*	Jelinic et al. [22]
	SCCOHT	100% (10 of 10)	Le Loarer et al. [88]
	SCCOHT	92% (24 of 26) *^c^*	Witkowski et al., 2014 [89]
	SCCOHT	91.9% (10 of 11) *^a^*	Jelinic et al. [90]
	SCCOHT	83.3% (15 of 18)	Lin et al. [91]
	SCCOHT	79% (19 of 24) *^a,c,d^*	Ramos et al. [25]
	SCCOHT	69% (9 of 13) *^a,c,d^*	Ramos et al. [92]
	SCCOHT	8 of 8 *^c^*	Moes-Sosnowska et al. [93]
	SCCOHT	7 of 7 *^a^*	Mazibrada et al. [94]
	SCCOHT	5 of 6	Auguste et al. [18]
	SCCOHT	2 of 2	Kupryjańczyk et al. [95]
	SCCOHT	2 of 2 *^a,c^*	Chandan et al. [96]
	SCCOHT	1 of 1 *^c^*	Sanders et al. [87]
	SCCOHT	1 of 1	Li et al. [97]
	SCCOHT	1 of 1	Gao et al. [98]
	SCCOHT	1 of 1 *^c^*	Pressey et al. [99]
	SCCOHT	1 of 1	Mathey et al. [100]
	SCCOHT	1 of 1 *^c^*	Mehta et al. [101]
	SCCOHT	1 of 1 *^c^*	Pastorczak et al. [102]
	SCCOHT	1 of 1 *^c^*	Connor et al. [103]
	SCCOHT	1 of 1 *^c^*	David et al. [104]
	SCCOHT	1 of 1	Bailey et al. [105]
	SCCOHT	1 of 1 *^a c^*	Lavrut et al. [106]
	SCCOHT	1 of 1 *^a^*	Fahiminiya et al. [107]
	SCCOHT	1 of 1 *^a^*	Gao et al. [108]
	SCCOHT	1 of 1 *^a^*	Aoyagi et al. [109]
			
	OCCC	5% (3 of 55)	Itamochi et al. [17]
			
	HGSOC	1 of 1 *^c^*	Muppala et al. [23]
*SMARCA2*	OCCC	2% (1 of 55)	Itamochi et al. [17]
*SMARCB1*	SCCOHT	1 of 1	Simões et al. [24]
	SCCOHT	1 of 1	Ramos et al. [25]
*SMARCC1*	OCCC	2% (1 of 55)	Itamochi et al. [17]

Overlap between cohorts exists in some studies. Percentages are reported for cohort sizes ≥ 10. Abbreviations: OCCC, ovarian clear cell carcinoma; EnOC, endometrioid ovarian cancer; MOC, mucinous ovarian cancer; HGSOC, high-grade serous ovarian cancer; SCCOHT, Small cell carcinoma of the ovary, hypercalcemic type. Symbols: *^a^* Corresponding immunohistochemical data are reported in Table 2; *^b^* with immature teratoma component; *^c^* includes patients with confirmed germline mutation; *^d^* extensive overlap exists between cases in these reports.

**Table 3:** Immunohistochemical analyses of specific SWI/SNF complex members identified in primary ovarian cancers of different histotypes.

Complex	Histotype	Loss of Expression	Reference
Member			
*ARID1A*	OCCC	76% (31 of 41) *^a^*	Kuroda et al. [15]
	OCCC	55% (23 of 42)	Yamamoto et al. [80]
	OCCC	39% (44 of 112)	Itamochi et al. [81]
	OCCC	33% (30 of 92)	Bennett et al. 2021 [27]
	OCCC	15% (9 of 60)	Katagiri et al. [82]
			
	EnOC	60% (12 of 20) *^a^*	Kuroda et al. [15]
			
	HGSOC	19% (7 of 36) *^a^*	Kuroda et al. [15]
	HGSOC	0% (0 of 17)	Katagiri et al. [82]
			
	MOC	0 of 6 *^a^*	Kuroda et al. [15]
*ARID1B*	OCCC	15% (8 of 53)	Sato et al. [85]
*SMARCA4*/	SCCOHT	100% (12 of 12)	Karianian-Phillipe et al. [110]
BRG1	SCCOHT	97% (34 of 25) *^a^*	Witkowski et al. [89]
	SCCOHT	96% (54 of 56) *^b^*	Clarke et al. [111]
	SCCOHT	94% (16 of 17)	Conlon et al. [112]
	SCCOHT	92% (46 of 50) *^c^*	Karnezis et al. [113]
	SCCOHT	89% (16 of 18)	Zheng et al. [114]
	SCCOHT	88% (39 of 44)	Genestie et al. [86]
	SCCOHT	84% (16 of 19) *^a^*	Ramos et al. [25]
	SCCOHT	82% (14 of 17) *^a^*	Ramos et al. [92]
	SCCOHT	64% (7 of 11) *^a,d^*	Jelinic et al. [90]
	SCCOHT	2 of 2 *^a^*	Chandan et al. [96]
	SCCOHT	1 of 1	Aggarwal et al. [115]
	SCCOHT	1 of 1 *^e^*	Atwi et al. [116]
	SCCOHT	1 of 1 *^a^*	Lavrut et al. [106]
	SCCOHT	1 of 1 *^a^*	Fahiminiya et al. [107]
	SCCOHT	1 of 1	Altmann et al. [117]
	SCCOHT	1 of 1 *^a^*	Gao et al. [108]
	SCCOHT	1 of 1	Saylany et al. [118]
	SCCOHT	1 of 1 *^a^*	Aoyagi et al. [109]
	SCCOHT	0 of 1	Coşkun et al. [119]
	SCCOHT	0 of 7 *^a^*	Mazibrada et al. [94]
			
	OCCC	5% (1 of 20)	Jelinic et al. [26]
	OCCC	4% (15 of 360)	Karnezis et al. [113]
	OCCC	3% (1 of 37)	Conlon et al. [112]
	OCCC	2% (2 of 93)	Ramos et al. [92]
	OCCC	0% (0 of 105)	Bennett et al. [27]
			
	HGSOC	0% (0 of 1198)	Karnezis et al. [113]
	HGSOC	0% (0 of 204)	Ramos et al. [92]
	HGSOC	0% (0 of 42)	Conlon et al. [112]
	HGSOC	0% (0 of 33)	Karianian-Phillipe et al. [110]
			
	endometrioid *^f^*	0% (0 of 268)	Karnezis et al. [113]
	EnOC	0% (0 of 38)	Conlon et al. [112]
	endometrioid *^f^*	0% (0 of 36)	Ramos et al. [92]
			
	mucinous *^g^*	0% (0 of 110)	Karnezis et al. [113]
	mucinous *^g^*	0% (0 of 14)	Ramos et al. [92]
			
	LGSOC	0% (0 of 53)	Karnezis et al. [113]
	LGSOC	(0 of 9)	Ramos et al. [92]
*SMARCA2*/	SCCOHT	100% (45 of 45) *^h^*	Karnezis et al. [113]
BRM	SCCOHT	90% (9 of 10)	Jelinic et al. [26]
	SCCOHT	86% (31 of 36)	Genestie et al. [86]
	SCCOHT	7 of 7 *^a^*	Mazibrada et al. [94]
	SCCOHT	5 of 6	Auguste et al. [18]
	SCCOHT	1 of 1	Sanders et al. [87]
	SCCOHT	1 of 1	Mehta et al. [101]
	SCCOHT	1 of 1	Simões et al. [24]
	SCCOHT	1 of 1	Altmann et al. [117]
			
	OCCC	8% (8 of 104)	Bennett et al. [27]
	OCCC	5% (1 of 20)	Jelinic et al. [26]
*SMARCB1*/	SCCOHT	13% (2 of 16)	Ramos et al. [25]
INI-1	SCCOHT	6% (3 of 50)	Karnezis et al. [113]
	SCCOHT	0% (37 of 37)	Clarke et al. [111]
	SCCOHT	0 of 1	Coşkun et al. [119]
	SCCOHT	0 of 1	Mehta et al. [101]
			
	OCCC	0% (0 of 150)	Bennett et al. [27]

Percentages are reported for cohort sizes ≥10. There is extensive overlap in cohorts studied in Ramos et al. [92], Ramos et al. [25], and Karnezis et al. [113]. Abbreviations: OCCC, ovarian clear cell carcinoma; EnOC, endometrioid ovarian cancer; MOC, mucinous ovarian cancer; HGSOC, high-grade serous ovarian cancer; SCCOHT, Small cell carcinoma of the ovary hypercalcemic type. Symbols: *^a^* Corresponding gene mutation data are reported in Table 1; *^b^* 41 samples in this study previously published, including genetic and immunohistochemical data; *^c^* SCCOHT cohort consists of 46 primary tumours, 2 patient-derived xenografts and 2 cell lines; *^d^* three samples reported as “equivocal” for BRG1/SMARCA4 immunostaining and one with an in-frame deletion showed the retention of BRG1/SMARCA4; *^e^* subsequent germline testing revealed a *SMARCA4* mutation; *^f^* all of endometrioid carcinoma, mixed carcinoma and borderline tumours; *^g^* both borderline and malignant mucinous tumours; *^h^* all tumours reported for loss of BRM/SMARCA2 immunostaining had a mutation in either *SMARCA4* (43 of 45) or *SMARCB1* (2 of 45).

A single study has reported additional SWI/SNF complex member mutations in a small number of OCCC cases, specifically in both of the mutually exclusive ATPase subunits *SMARCA4* (3 of 55 tumours) and *SMARCA2* (1 of 55 tumours), as well as in the core scaffold subunit *SMARCC1* (1 of 55 tumours) (Table 2) [17]. Furthermore, the loss of SMARCA4 detected by immunohistochemistry has been reported in between 2–5% of OCCC [26,92,112,113]. The immunohistochemical loss of SMARCA2 has also been reported in OCCC in between 5–8% of cases [26,27]. The dual loss of SMARCA4 and SMARCA2 has not been reported in OCCC [26,27]. A single immunohistochemical study of SMARCB1 in 105 OCCCs showed the retention of this protein [27]. It will be interesting to determine whether additional mutations or otherwise dysregulated SWI/SNF complex members will be identified in new studies of primary OCCCs.

### 3.2. Small Cell Carcinoma of the Ovary, Hypercalcaemic Type (SCCOHT)

#### 3.2.1. Clinical Presentation and Epidemiology of SCCOHT

SCCOHT is an exceedingly rare and aggressive subtype of ovarian cancer, believed to account for less than 0.01% of all ovarian malignancies [120]. Cells constituting primary SCCOHT tumours have been described as small and round with hyperchromatic nuclei and minimal cytoplasm [121]. The cell of origin has been under debate, although aspects of its clinical presentation that include positive staining for the germ-cell markers SALL4, OCT3/4, alpha-fetoprotein (AFP) and glypican 3 suggest that SCCOHT is most likely of germ-cell origin [122]. Similarities between SCCOHT and malignant rhabdoid tumours have been drawn, with one study suggesting that SCCOHT be renamed as ‘malignant rhabdoid tumour of the ovary’ [123].

In contrast to other subtypes of ovarian cancer that generally, but not always, occur in post-menopausal women, SCCOHT is predominantly diagnosed in the vastly different demographic of infants, children and women of child-bearing age, with a median age of onset of 25 years [8,124]. The youngest diagnosis of SCCOHT reported to date is in a 12-month-old child, while the oldest is a woman of 56 years [116,125]. A recent case report described a 28-year-old woman diagnosed in the last trimester of pregnancy [118]. Patients have reported with general symptoms including nausea, weight loss, constipation and fatigue, as well as abdominal pain and/or swelling. Between 50–70% of patients with SCCOHT have associated hypercalcaemia [8,126,127]. Immunohistochemical analyses of primary tumours have observed elevated parathyroid hormone-related protein (PTHrp) in some cases, suggesting that PTHrp may be the underlying cause of hypercalcaemia in SCCOHT [128]. In a systematic review of 67 studies describing 306 SCCOHT patients, elevation of the serum glycoprotein CA-125 was reported in around 80% [8].

The five-year OS for patients diagnosed at the earliest stage (FIGO Stage I) has been reported as 51%, with patients diagnosed after this time (FIGO Stages II-IV) having a reduced OS of only 24% [8]. Both familial and sporadic cases of SCCOHT have been reported [124,125]. The international SCCOHT consortium has proposed consensus guidelines for the diagnosis and care of SCCOHT patients, including radical surgery, adjuvant chemotherapy and radiotherapy [121]. Despite this, the risk of spread beyond the ovary remains high and outcomes are poor [127]. While SCCOHT tumours are initially chemosensitive, the time to recurrence can be short, with relapsed tumours displaying a reduced response to chemotherapy [121,127].

#### 3.2.2. SWI/SNF Complex Member Mutations in SCCOHT

SCCOHT tumours have a low tumour mutational burden (TMB), with studies reporting less than six mutations/Mb on a background of genomic stability and low copy number changes [18,91]. The mutation of one of the SWI/SNF ATPases, SMARCA4, is the predominant mutation in SCCOHT, reported in between 69–100% of cases in studies of 10 or more patients [22,25,88,89,90,91,92]. Similar results have been reported in studies of less than 10 patients, as well as in many case studies [18,87,93,94,95,96,97,98,99,100,101,102,103,104,105,106,107,108,109]. Numerous studies have reported the presence of a germline *SMARCA4* mutation in SCCOHT [22,25,87,89,92,93,96,99,101,102,103,104,106]. This would indicate that at least in these tumours, *SMARCA4* mutation is occurring as an early event across the timeline of tumorigenesis. *SMARCA4* mutation has also been implied by immunohistochemical studies of SMARCA4 depletion in the absence of genetic analyses [86,110,111,112,113,114,115,116,117,118,119]. Reports of *SMARCA4* mutations and protein loss in SCCOHT are summarised in Table 2 and Table 3.

The loss or depletion of SMARCA2, but not *SMARCA2* mutation, is reported in SCCOHT concomitant with *SMARCA4* mutations, implying epigenetic mechanisms of SMARCA2 silencing [18,24,26,86,87,94,101,113,117] (Table 2 and Table 3). The loss of both the SWI/SNF ATPases is exceedingly rare in human malignancy, as cancer cells normally need at least one of these ATPases to be functioning in order to survive, proliferate and metastasise [113]. Rare variants of non-ovarian cases of concomitant SMARCA2 and SMARCA4 loss have been reported for cancer types including lung [88,129], endometrial [130], gastrointestinal tract [131], sinonasal undifferentiated carcinoma [132], and rhabdoid tumours [133]. The key survival mechanisms of this intriguing dual loss of the mutually exclusive SWI/SNF ATPases SMARCA4 and SMARCA2 remain to be determined. It has been reported that after the dual loss of SMARCA4 and SMARCA2, the residual SWI/SNF complex can still bind to accessible chromatin but results in disrupted transcriptional regulation that impacts upon the expression of genes involved in cellular processes and behaviours, including differentiation, epithelial–mesenchymal transition (EMT), metastasis, DNA repair, apoptosis, adhesion, immunity, metabolism, drug metabolism, proliferation and angiogenesis [134,135].

The mutation of SMARCB1, one of the most conserved subunits of SWI/SNF, is rarely reported in SCCOHT. A 19-year-old woman presented with a highly aggressive case of SCCOHT that was found to have a somatic nonsense point mutation in *SMARCB1* with loss of the wild-type allele and a low TMB of less than two mutations/Mb [24]. The patient received high-dose chemotherapy and stem cell transplantation; however, disease progression was rapid and she died 11 months after the presentation of initial symptoms. A homozygous frameshift *SMARCB1* mutation in a SCCOHT tumour has also been reported [25] (Table 2). The immunohistochemical loss of SMARCB1 has been reported in 6% (3 of 50) [113] and 13% (2 of 16) [25] of SCCOHT tumours studied (Table 3). Mutation of *ARID1A* and *ARID1B* has also been infrequently reported in SCCOHT [18,86,87] (Table 2).

### 3.3. Endometrioid Ovarian Cancer (EnOC) and Other Ovarian Cancer Subtypes—Links to Abrogated SWI/SNF

Similar to OCCC, EnOC has also been associated with the presence of endometriosis, with these two subtypes together referred to as EAOCs [14,19,20,21,56]. A large systematic review and meta-analysis found a 2.3-fold higher risk of EnOC in women with endometriosis [136]. EnOC is a distinct ovarian cancer subtype accounting for up to ~15% of all ovarian cancers [137]. It tends to be diagnosed at an earlier stage (FIGO Stages I–II), and as a result, most patients have a more favourable prognosis than other ovarian cancer subtypes [138]. In addition to its association with endometriosis, EnOC is also associated with Lynch syndrome [139] and can occur in patients who have endometrial cancer [138,140]. Relative to SCCOHT and OCCC, there are fewer reports investigating SWI/SNF complex members in EnOC. *ARID1A* mutations have been reported in between 21–45% of cases of EnOC [14,15,74,79] (Table 2). Furthermore, the immunohistochemical loss of ARID1A has been reported in 60% (12 of 20) of EnOC cases [15]. In studies of EnOC, mixed carcinoma and borderline tumours did not identify the immunohistochemical loss of SMARCA4 (Table 3) [92,112,113].

Table 2 and Table 3 also summarise the involvement of SWI/SNF complex members in ovarian cancer subtypes other than OCCC, EnOC and SCCOHT. *ARID1A* mutations have been reported in a single study in 2 of 6 cases of MOC [15]; however, the same study did not report a corresponding loss of ARID1A following immunohistochemical analyses. Further, correlations observed between *ARID1A* mutations and CD8 and PD-L1 (programmed death ligand 1) levels in other ovarian cancer subtypes were not observed for MOC in this study [15]. Immunohistochemical studies of borderline and malignant MOC have not shown a loss of SMARCA4 [92,113]. Two immunohistochemical studies in low-grade serous ovarian cancer (LGSOC) reported a retention of SMARCA4 [92,113]. Of note, mutation of *ARID1A* has been reported infrequently in HGSOC [14,15,16], with a single case report of a *SMARCA4* mutation in HGSOC [23]. This patient presented at 57 years of age, and her *SMARCA4* mutation was found to be germline. It is important to note that this patient also had a *BRCA2* variant of unknown significance that could not be excluded as a driver mutation of her HGSOC. Further, SMARCC1, referred to as BAF155, is proposed to be methylated by the arginine methyltransferase CARM1 (Coactivator-Associated Arginine Methyltransferase 1) in HGSOC, typically without mutations in *BRCA1* or *BRCA2* [141]. While interesting, additional studies of CARM1 amplification and BAF155 methylation leading to down-regulation in primary ovarian tumours would be required to draw further conclusions.

### 3.4. Endometriosis and the SWI/SNF Complex

Globally, endometriosis is believed to affect 5–10% of women of reproductive age [142], however, this figure increases for certain groups of patients, such as those who are symptomatic [143]. Despite these high frequencies, the exact cause of this chronic condition has not been determined, with theories including retrograde menstruation and endometriotic lesions being of stem cell origin [144]. Numerous studies have reported an elevated risk of developing ovarian cancer in patients with endometriosis [64,66,67,145,146,147,148,149,150,151]. The most recent and one of the largest of these studies by Barnard and colleagues investigated the Utah Population Database and reported a 4.2-fold higher ovarian cancer risk in women with endometriosis compared to those without, and a 9.7-fold greater risk for women with ovarian endometriomas and/or deep-infiltrating endometriosis [145]. Similar results were also seen in an earlier study, whereby a higher proportion of these ovarian cancers were OCCC or EnOC [136]. Endometriosis has been identified in between 21–51% of women with OCCC and 23–43% of women with EnOC [146,152,153].

Identical mutations in *ARID1A* have been identified in OCCC and synchronous endometriotic lesions, providing a strong argument for malignant OCCC arising from these apparently benign lesions [67]. Should this be the case, it would suggest that at least in OCCC associated with endometriosis, the loss of *ARID1A* is an early event in the timeline of tumorigenesis. Endometriotic lesions occurring without EAOCs or distant from an ARID1A-deficient EAOC have shown diffuse immunoreactivity for ARID1A [80]. The immunohistochemical loss of ARID1A in endometriotic lesions has been suggested as a putative prognostic biomarker for ovarian cancer risk [149]. The potential involvement of other abrogated SWI/SNF complex members in endometriosis is currently unknown. The underlying factors driving the malignant transformation of endometriotic lesions remain to be fully elucidated, with suggestions including exposure to oestrogen or an imbalance of oestrogen receptors, oxidative stress, inflammatory processes, local nutrient availability and metabolic reprogramming [19]. Whether ARID1A defects present an actionable target in endometriosis also remains to be determined, with the types of drugs discovered to treat malignancy needing to be considered in a different context for potential management of a predominantly benign chronic condition.

## 4. SWI/SNF Abrogation in Ovarian Cancer Represents Therapeutic Vulnerabilities

**Figure 2:**
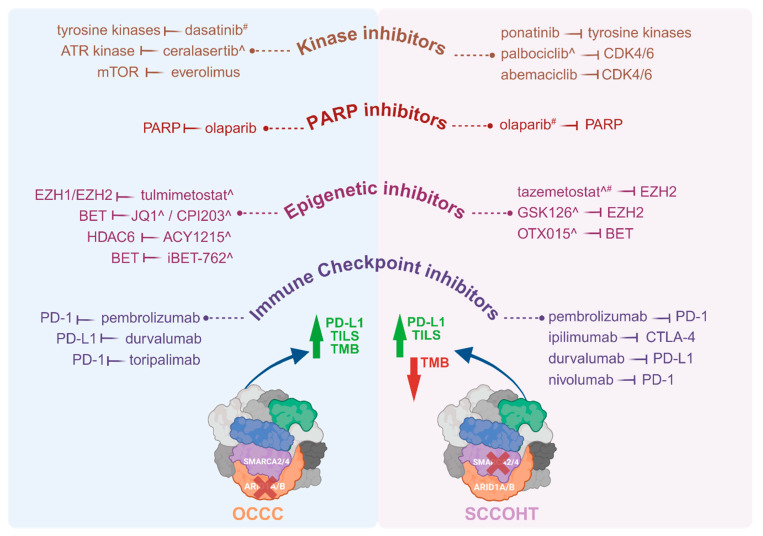
Therapeutic drugs investigated in patients with OCCC or SCCOHT and pre-clinical models of these tumours. Molecular targeted therapies including immune checkpoint inhibitors, epigenetic inhibitors, PARP inhibitors and kinase inhibitors have been trialled in patients with OCCC or SCCOHT, as well as in vitro and in vivo pre-clinical models ^ of these malignancies. Where patients did not respond to, or tolerate, a drug, this is indicated by ^#^. Drugs listed were administered to patients or tested in pre-clinical models either as monotherapies or in conjunction with other drug(s). A higher TMB is reported for OCCC, indicated by a green arrow, while SCCOHT has a low TMB, indicated by a red arrow. Both OCCC and SCCOHT have TILs. Both tumour types have high levels of PD-L1. Therapeutic drugs tested in OCCC patients, include pembrolizumab [154], durvalumab [155], toripalimab [156], olaparib [157], everolimus [156], and dasatinib [158], and in pre-clinical models include iBET-762 [159], ACY1215 [160], CPI203 [159], ceralasertib [161] and tulmimetostat [162]. Therapeutic drugs tested in SCCOHT patients include nivolumab [163], ipilimumab [163], pembrolizumab [103,164], durvalumab [108], olaparib [108,163], tazemetostat [165], abemaciclib [163], palbociclib [108] and ponatinib [163], and in pre-clinical models include GSK126 [166], OTX015 [167], tazemetostat [166,168] and palbociclib [169]. Drugs trialled in patients were on occasion administered either sequentially, informed by patient response, or together. Drug combinations of this nature in OCCC patients included pembrolizumab (combined with bevacizumab and cyclophosphamide) [154], pembrolizumab (combined with bevacizumab and olaparib) [157], and toripalimab (combined with everolimus) [156]. Drug combinations trialled in SCCOHT patients included pembrolizumab (following cycles of cisplatin/etoposide and carboplatin/paclitaxel) [103], nivolumab and ipilimumab (followed by ponatinib, abemaciclib and olaparib) [163]. In a single case report, a SCCOHT patient was administered six lines of chemotherapy of multiple drugs that included durvalumab, olaparib and palbociclib [108]. Abbreviations: ATR, Ataxia-telangiectasia-mutated (ATM) and RAD3-related; BET, bromo- and extra-terminal domain family; CDK4/6, cyclin-dependent kinases 4 and 6; CTLA-4, cytotoxic T-lymphocyte-associated protein 4; EZH1/2, Enhancer Of Zeste 1/2 Polycomb repressive complex 2 subunit; HDAC6, histone deacetylase 6; mTOR, mammalian target of rapamycin; OCCC, ovarian clear cell carcinoma; PARP, Poly (ADP-ribose) polymerase; PD-1, programmed cell death protein 1; PD-L1, programmed death-ligand 1; SCCOHT, Small cell carcinoma of the ovary, hypercalcaemic type; TILS, Tumour-infiltrating lymphocytes; TMB, tumour mutational burden. Both tumour types also have high levels of PD-L1. Created with www.BioRender.com, Access Date: 9 August 2024.

Pharmacological targeting of the effects of mutant SWI/SNF complex members, with a specific focus on mutant ARID1A and SMARCA4 for the purpose of treating patients with OCCC or SCCOHT, span a number of drug classes. These include immunotherapeutics, kinase inhibitors, inhibitors of the DNA damage response including PARP inhibitors, and epigenetic inhibitors (Figure 2).

### 4.1. Immunotherapy

Most ovarian cancer subtypes, and especially the most frequently diagnosed HGSOC, at best show only modest responses to immunotherapy, with clinical trials suggesting responses as low as 8% of patients [170,171,172,173]. This does not appear to be the case for OCCC and SCCOHT, with ICB being trialled on patients with SCCOHT and OCCC. The knockout of *ARID1A* has been shown to increase levels of *CD274* and the protein it encodes—PD-L1, the predominant ligand of programmed death 1 (PD-1)—both in vitro and in vivo [160,174]. Wild-type ARID1A and ARID1B are located at the promoter of *CD274* in OCCC cells (shown in the human OVCA429 and RMG1 cell lines, as well as mouse ovarian ID8-Defb29/Vegf cancer cells), although ARID1B was not able to influence the levels of CD274 in *ARID1A* knockout (KO) cells [160]. CRISPR-Cas9 KO of *ARID1A* in ID8 cells investigated in both an intraperitoneal and an orthotopic model showed increased tumour-infiltrating lymphocytes and PD-L1 levels and a greater response to an anti-PD-L1 antibody compared to WT *ARID1A* tumours, with lower tumour burden and prolonged survival [174]. All of these factors suggest that a response to ICB is likely in the presence of *ARID1A* mutation.

ARID1A is a binding partner of the mismatch repair (MMR) protein MSH2, although it does not appear to regulate *MSH2*, or other MMR genes (*MLH1*, *MSH3*, *MSH6*, *PMS1* and *PMS2*), at the transcriptional level [174]. OCCC has a higher TMB than other ovarian cancer subtypes, although this is not seen in all cases [15,175]. Like SCCOHT, OCCC has a relatively low level of copy number abnormalities, possibly due to the loss of ARID1A causing defects in telomere cohesion leading to the removal of defective chromosomal changes during mitosis [176]. The anti-PD-L1 monoclonal antibody durvalumab that blocks the binding of PD-L1 and PD-1, as well as CD80, has been trialled in OCCC (www.ClinicalTrials.gov, NCT03405454) [155]. A combination of pembrolizumab (anti-PD-1 monoclonal antibody), bevacizumab (anti-angiogenic anti-VEGF monoclonal antibody) and cyclophosphamide has been trialled in a small number of patients with OCCC, with durable responses observed, but also treatment-limiting toxicities [154]. ICB has been the focus of other studies in OCCC with mixed results in individual patients [156,177]. Investigation of an OCCC molecular signature that maximises the likelihood that a patient will respond to ICB is warranted [178].

In contrast to OCCC, SCCOHT tumours have low TMB and are genomically stable, but like OCCC have low copy number variations [18,91]. This molecular profile would suggest that SCCOHT may not be responsive to immunotherapy; however, many tumours have been shown to have high PD-L1 expression in both tumour and stromal cells accompanied by robust associated T-cell infiltration [90]. It is possible that the loss of SMARCA4 in SCCOHT reprograms the transcriptional landscape to influence tumour immunogenicity, creating an environment that is more permissive to ICB [90].

Anti-PD-1 ICB with pembrolizumab has been trialled in SCCOHT patients with encouraging results [90,164], although not all SCCOHT patients respond to ICB [103,164]. A 34-year-old patient diagnosed with FIGO Stage II SCCOHT with a somatic *SMARCA4* mutation was reported to have a remarkable response of over 5 years of survival post recurrence, treated with ICB plus anti-angiogenic therapy and CDK4/6 (cyclin-dependent kinase 4 and 6) inhibitors [108]. A 21-year old patient diagnosed with SCCOHT and a germline *SMARCA4* mutation underwent high-dose chemotherapy followed by an autologous stem cell transplant and a combination of drugs, including nivolumab (anti-PD-1 monoclonal antibody) and ipilimumab (anti-CTLA-4 (Cytotoxic T-Lymphocyte Associated Protein 4) monoclonal antibody), as well as the multi-tyrosine kinase inhibitor ponatinib and abemaciclib (selective CDK4/6 inhibitor), surviving at least three years post diagnosis [163]. Clinical trial activity focused on ICB for SCCOHT patients, including in combination with other therapies, have been undertaken or are currently active [108,121]. As for OCCC, decisions regarding ICB in SCCOHT patients will benefit from determining molecular signatures that are congruent with response to ICB drugs.

### 4.2. Kinase Inhibitors

Drugs in the receptor tyrosine kinase family have been shown to have selective benefit in SCCOHT cell lines with the dual loss of SMARCA4 and SMARCA2 [179]. Unbiased synthetic lethal screens using a short hairpin RNA (shRNA) targeting the human kinome in a SMARCA4/SMARCA2-deficient model of SCCOHT led to the identification of CDK4/6 inhibitors as a molecular target therapy for this malignancy [169]. The molecular mechanism underpinning this sensitivity is low levels of cyclin D1 in SMARCA4-deficient cells, given that SMARCA4 directly regulates the transcription of the cyclin D1 gene *CCND1* [169]. As noted above, treatment responses in SCCOHT have been observed with ponatinib and abemaciclib in conjunction with other therapies, including ICB [163]. Dasatinib, a second-generation multi-tyrosine kinase inhibitor administered as a monotherapy, did not show treatment benefits for OCCC [158]. The loss of ARID1A has been linked to the activation of MAPK signalling [180], sensitivity to PI3K/AKT/mTOR inhibitors and ATR inhibitors [161,181], as well as aurora kinase inhibition [182], warranting further investigation for the development of molecularly targeted therapies against *ARID1A*-mutated ovarian cancers.

### 4.3. PARP Inhibitors

The SWI/SNF complex plays an active role in modelling the accessibility of chromatin to accommodate complexities of the DNA damage response [38,183]. In cells with intact SWI/SNF, ARID1A is recruited to DSBs through its association with the DNA damage checkpoint kinase ATR [174]. In cells lacking ARID1A, there is a delay in the recruitment of repair factors to sites of DNA damage. Further, in ARID1A null cells, increased PARP activity has been reported, making these cells susceptible to PARP inhibition with olaparib, and especially in conjunction with ionising radiation therapy [181]. This increased sensitivity of ARID1A null cells to PARP inhibition is seen both in vitro and in vivo [174]. Some OCCC cell lines are sensitive to PARP inhibitors in vitro [184]. The alkylating agent temozolomide (TMZ) combined with PARP inhibition has also been shown to induce replication fork instability and apoptosis in *ARID1A* mutant ovarian cancer xenografts [185]. An OCCC patient with an *ARID1A* mutated tumour achieved a partial and sustained response to the combination of olaparib, pembrolizumab and bevacizumab [157]. A compelling case for the use of PARP inhibitors in SCCOHT is yet to be made, given that olaparib is not always well tolerated in these patients [108,163].

### 4.4. Epigenetic Inhibitors

The interaction of the SWI/SNF complex with other epigenetic complexes and regulators offers opportunities for therapeutic interventions based on synthetic lethal relationships with mutant SWI/SNF complex members. To date, the exploration of epigenetic inhibitors to treat either OCCC or SCCOHT has been predominantly confined to pre-clinical models only. In this vein, the antagonistic relationship of SWI/SNF and the PRC2 has raised the pharmacological inhibition of the PRC2 catalytic subunit EZH2, a histone methyltransferase, in *ARID1A* mutant cells, including OCCC, as a potential therapy [186,187,188]. The second-generation EZH2 inhibitor tulmimetostat has shown promise in pre-clinical models of *ARID1A* mutated malignancies, including OCCC [162]. EZH2 inhibition is also under investigation for the treatment of SCCOHT. The EZH2 inhibitor tazemetostat (EPZ-6438) has displayed efficacy in pre-clinical models of SCCOHT with SMARCA4/SMARCA2 loss [166,168], as has the EZH2 inhibitor GSK126 [166]. It remains to be determined whether strong responses in SCCOHT patients will be generally achieved [189], however, a Phase I/II trial for tazemetostat (NCT02601950) failed stage 2 futility that needed a confirmed partial response or complete response in at least five patients based on the RECIST 1.1 criteria [165].

Preclinical models of SCCOHT have also demonstrated responses to pan-HDAC inhibitors, including quisinostat, and in combination with EZH2 inhibitors at sub-lethal doses have worked synergistically to induce apoptosis both in vitro and in vivo [189]. Using the same strategies of targeting mutant SWI/SNF, the HDAC6 inhibitor ACY1215 combined with anti-PD-L1 antibody reduced tumour burden and eliminated ascites in an in vivo model of ARID1A-inactivated OCCC [160]. Lastly, the BET (bromodomain and extra-terminal domain) family of proteins classified as epigenetic readers is being considered for inhibition in both OCCC and SCCOHT. BET inhibitors JQ1 and iBET-762 have shown efficacy in in vitro and in vivo models of *ARID1A* mutant OCCC [159]. Combination drug treatments with the BET inhibitor CPI203 and select PI3K-AKT inhibitors showed efficacy in preclinical models of OCCC, although this appeared to be independent of *ARID1A* mutation status [190]. Combinations of the BET inhibitor OTX015 that targets BET family member BRD2 (bromodomain containing 2) and MEK (mitogen-activated protein kinase kinase) inhibitors have also shown efficacy in preclinical models of SCCOHT, although this does not appear to be specific for tumours with concomitant SMARCA4 and SMARCA2 loss [167].

## 5. Conclusions

Mutation and epigenetic silencing of SWI/SNF complex members are associated with the poorer prognosis, chemoresistant subtypes of ovarian cancer occurring in younger patients. In the case of SCCOHT, this disease can occur in infants. *ARID1A* mutation is present in endometriosis, and given that OCCC and EnOC are identified as EAOCs, this points to the involvement of abrogated SWI/SNF as an early driver of tumorigenesis. Studies investigating the mechanistic behaviour of mutant SWI/SNF complex members are revealing potential therapeutic opportunities for the treatment of patients with OCCC and SCCOHT that have already demonstrated clinical benefit for some patients. A deeper understanding of mutant SWI/SNF in ovarian and other cancers, as well as in endometriosis, may reveal new opportunities for the pharmacological inhibition of the key molecular targets associated with these diseases.

## Figures and Tables

**Table 1:** Nomenclature for SWI/SNF (BAF) complex members mutated or otherwise lost in ovarian cancer subtypes ^.

Gene Name	Protein Names	Function/Role
*ARID1A*	ARID1A, BAF250A	DNA binding subunit, paralogue of ARID1B
*ARID1B*	ARID1B, BAF250B	DNA binding subunit, paralogue of ARID1A
*SMARCA2*	SMARCA2, BRM	mutually exclusive ATPase subunit
*SMARCA4*	SMARCA4, BRG1	mutually exclusive ATPase subunit
*SMARCB1*	SMARCB1, BAF47, hSNF5, INI-1	core complex member binding directly to acidic patch on nucleosomes
*SMARCC1*	SMARCC1, BAF155, SRG3	core complex member promoting complex stability

^ nomenclature used by referenced studies (for an exhaustive list of aliases, see Genecards https://www.genecards.org, Access Date: 9 August 2024). Abbreviations: ARID1A (AT-rich interactive domain-containing protein 1A); ARID1B (AT-rich interactive domain-containing protein 1B); BAF (BRG1-or BRM-associated factors); BAF47 (BRG1-associated factor 47); BAF155 (BRG1/BRM-associated factor 155); BAF250A (BRG/BRM-associated factor A); BAF250B (BRG/BRM-associated factor B); BRG1 (Brahma-related gene 1); BRM (Brahma); hSNF5 (human Sucrose Non-Fermenting 5); INI-1 (Integrase Interactor 1); SMARCA2 (SWI/SNF-Related, Matrix-Associated, Actin-Dependent Regulator of Chromatin, Subfamily A, Member 2); SMARCA4 (SWI/SNF-Related, Matrix-Associated, Actin-Dependent Regulator of Chromatin, Subfamily A, Member 4); SMARCB1 (SWI/SNF-Related, Matrix-Associated, Actin-Dependent Regulator of Chromatin, Subfamily B, Member 1); SMARCC1 (SWI/SNF-Related, Matrix-Associated, Actin-Dependent Regulator of Chromatin Subfamily C Member 1); SRG3 (SWI/SNF-related gene 3); SWI/SNF (SWItch/Sucrose Non-Fermentable; also known as the BAF complex).

## Data Availability

Not applicable.
